# The prognostic value of schizontaemia in imported *Plasmodium falciparum* malaria

**DOI:** 10.1186/1475-2875-11-301

**Published:** 2012-08-28

**Authors:** Marlies E van Wolfswinkel, Mariana de Mendonça Melo, Klaske Vliegenthart-Jongbloed, Rob Koelewijn, Jaap J van Hellemond, Perry J van Genderen

**Affiliations:** 1 Department of Internal Medicine, Harbour Hospital, Rotterdam, The Netherlands; 2 Institute for Tropical Diseases, Harbour Hospital, Haringvliet 72, 3011, TG, Rotterdam, The Netherlands; 3 Laboratory of Parasitology, Harbour Hospital and Institute for Tropical Diseases, Rotterdam, The Netherlands; 4 Department of Medical Microbiology and Infectious Diseases, Erasmus Medical Centre, Rotterdam, The Netherlands

**Keywords:** Schizont, Prognosis, Malaria, Falciparum, Import, Severe

## Abstract

**Background:**

In *Plasmodium falciparum* infection, peripheral parasite counts do not always correlate well with the sequestered parasite burden. As erythrocytes parasitized with mature trophozoites and schizonts have a high tendency to adhere to the microvascular endothelium, they are often absent in peripheral blood samples. The appearance of schizonts in peripheral blood smears is thought to be a marker of high sequestered parasite burden and severe disease. In the present study, the value of schizontaemia as an early marker for severe disease in non-immune individuals with imported malaria was evaluated.

**Methods:**

All patients in the Rotterdam Malaria Cohort diagnosed with *P. falciparum* malaria between 1 January 1999 and 1 January 2012 were included. Thick and thin blood films were examined for the presence of schizontaemia. The occurrence of WHO defined severe malaria was the primary endpoint. The diagnostic performance of schizontaemia was compared with previously evaluated biomarkers C-reactive protein and lactate.

**Results:**

Schizonts were present on admission in 49 of 401 (12.2%) patients. Patients with schizontaemia were more likely to present with severe malaria, a more complicated course and had longer duration of admission in hospital. Schizontaemia had a specificity of 0.95, a sensitivity of 0.53, a negative predictive value of 0.92 and a positive predictive value of 0.67 for severe malaria. The presence of schizonts was an independent predictor for severe malaria.

**Conclusion:**

Absence of schizonts was found to be a specific marker for exclusion of severe malaria. Presence of schizonts on admission was associated with a high positive predictive value for severe malaria. This may be of help to identify patients who are at risk of a more severe course than would be expected when considering peripheral parasitaemia alone.

## Background

Sequestration of infected erythrocytes in the microvasculature is one of the main pathological features of severe *Plasmodium falciparum* infection [1-4]. Particularly, erythrocytes parasitized with mature trophozoites and schizonts have a high tendency to adhere to the microvascular endothelium and are therefore often absent in collected peripheral blood samples. However, the appearance of schizonts in peripheral blood smears is thought to reflect a high sequestered parasite burden and was recognized as a sign of severe disease already in early field studies on malaria [5]. The predominance of mature parasites on peripheral smears was found to reflect more severe disease by Silamut and White [6], but only a few other studies on the performance of schizontaemia as a predictor for severe disease have been done [7-9]. In addition, data is only available from studies in areas with malaria endemicity where many inhabitants are believed to have some degree of immunity toward malaria (so-called semi-immunity). Data in non-immune individuals are lacking.

Although it is stated in the current WHO malaria guidelines that the presence of schizontaemia may be associated with a poor outcome in *P. falciparum* malaria, presence of schizonts is currently not a well-established WHO criterion for severe disease [9,10]. To further explore the value of schizontaemia as an early marker for severe disease in non-immune individuals with imported malaria, the prognostic value of schizontaemia was assessed in a large cohort of travellers with *P. falciparum* malaria.

## Methods

### Patients

The Harbour Hospital is a 161-bed general hospital located in Rotterdam, The Netherlands. It comprises the Institute for Tropical Diseases, which serves as a national referral centre. All patients in the Rotterdam Malaria Cohort diagnosed with *P. falciparum* malaria between 1 January 1999 and 1 January 2012 were included in this observational study. Of all patients identified, demographic, clinical and laboratory data were collected using a standardized form and stored in an electronic database. Patients with a mixed infection of *P. falciparum* with another *Plasmodium* species were included in this study, but pure non-falciparum *Plasmodium* infections were excluded. Multiple malaria episodes in a single patient were only regarded as separate cases if caused by true re-infection; recrudescent infections of *P. falciparum* were excluded.

### Laboratory investigations

Available laboratory examinations included red and white blood cell counts, haematocrit, platelet counts, C-reactive protein, serum electrolytes, total bilirubin, serum creatinine and urea, sodium, potassium, liver enzymes, blood glucose and venous plasma lactate.

### Detection of *Plasmodium* parasites

The standard procedure to diagnose malaria comprised a quantitative buffy coat (QBC) analysis, a rapid diagnostic test (RDT) for malaria antigens Binax NOW® Malaria Test (Binax, Inc, Maine, USA), and thick and thin blood smears using freshly collected blood specimens from finger pricks. The malaria rapid test and the QBC analysis were performed according to the manufacturer’s instructions. QBC capillaries were examined independently by two technicians by microscopic analysis of two complete rows of the region between the bottom of the capillary and the polynuclear leukocyte layer using an Olympus BX-60 fluorescence microscope equipped UV-filter and 50x objective and 12.5x oculars (total magnification 625x). These two lanes represented about 100 microscopic fields (at 625x magnification) and took an average examination time of 5 min. Subsequently, the polynuclear and mononuclear cell layer was screened for schizonts, gametocytes, malaria pigment and elderly trophozoites of *Plasmodium vivax* and *Plasmodium ovale*.

Thick blood smears were stained with Field’s stain (Brunschwig Chemie, Amsterdam, The Netherlands) and thin smears with Diff Quick stain (Medion Diagnostics, Düdingen, Switzerland). Both staining procedures had been optimized for optimal staining of *Plasmodium* parasites as well as Schüffner’s dots and Maurer’s clefts in infected erythrocytes. Thick and thin smears were examined with regular light microscopes at a total magnification of 1,250x. The *Plasmodium* species was identified on morphological characteristics in the thin and thick blood smears. Since a real-time quantitative PCR became available several years ago, a substantial proportion of the samples were additionally confirmed by PCR (without discrepant findings compared to classic light microscopy).

### Parasite counting and identification of *Plasmodium falciparum* schizonts

Parasitaemia was determined by parasite counting as described before [11]. *Plasmodium falciparum* schizonts were defined as clustered malaria pigment adjacent to two or more nuclei of *P. falciparum* in thick blood smears or QBC capillaries. Compared to thin and thick blood smears QBC analysis is the most sensitive test to detect *Plasmodium* parasites. *Plasmodium falciparum* schizonts can be identified in the erythrocyte layer in the QBC capillary, but identification within the leukocyte layer is difficult due to the intense fluorescent signal of the leukocyte nuclei. For this reason, this layer is also screened for quenched fluorescence by malaria pigment. If malaria pigment was detected in the leukocyte layer of the QBC capillary, the thick and thin blood smears were thoroughly examined for the presence of schizonts by analysis of at least 200 microscopic fields at 625x magnification.

## Definitions

### Severe malaria

Patients were classified as having severe malaria if they met one or more of the WHO criteria for severe malaria, as published [9], either on presentation or later during hospital admission.

### Schizontaemia

Schizontaemia was defined as presence of one or more *P. falciparum* schizonts identified in QBC and/or blood smear examinations from a blood sample taken on admission.

### Immunity to malaria

The degree of immunity to malaria was estimated as previously described [12]. In brief, adult immigrants from a malaria-endemic country living in The Netherlands were considered partially immune. Immigrant patients who had been living in a malaria-endemic area for at least two years at the time of diagnosis were presumed semi-immune. Tourists from non-endemic countries who travelled to malaria-endemic regions were considered non-immune.

### Statistical analysis

All data were not normally distributed (Kolmogorov-Smirnov test) and are therefore presented as medians and range. Univariate comparisons were performed using the Mann–Whitney *U* test and the Fisher’s Exact test. Trends between categorical variables were done with the Chi square test for Trend. Odds ratios were calculated using the approximation of Woolf. For clinical reference, the diagnostic performance of schizontaemia for severe disease was compared to that of the classic biomarkers serum C-reactive protein and plasma lactate [13-15]. Binary logistic regression analysis was used to analyse if schizontaemia was independently associated with malaria severity. Malaria severity was entered as the dependent variable. To test its independence from parasite load and the previously evaluated markers lactate and C-reactive protein, these were entered as co-variants. As parasite load was a highly skewed continuous variable with a logarithmic distribution, the data were transformed to log-parasitaemia to make them suitable for logistic regression analysis.

## Results

### Patient characteristics

Between 1 January 1999 and 1 January 2012, a total of 562 cases of imported malaria were seen. Of these cases, 161 cases were caused by non-falciparum *Plasmodium* species; 401 infections were caused *P. falciparum* infections including three patients who had a mixed infection (two with *P. falciparum* and *P. ovale*, one with *P. falciparum* and *P. vivax*, respectively). No patient with a mixed *P. falciparum* infection presented with schizontaemia on admission. These 401 patients with either a pure *P. falciparum* infection or a mixed *Plasmodium* infection with *P. falciparum* were included in this study.

### Schizontaemia

**Figure 1:**
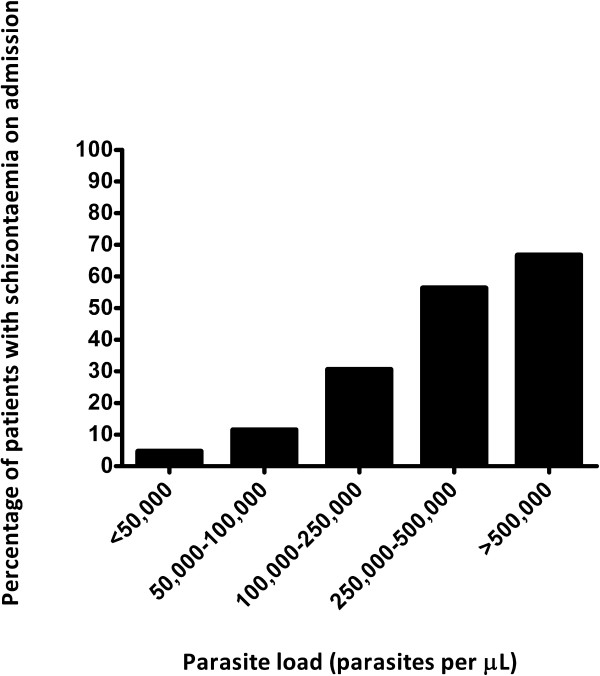
**The percentage of patients with schizontaemia on admission, stratified by peripheral parasite count**.

Schizonts were present on admission in 49 of 401 (12.2%) patients. The general characteristics of the patients with presence and absence of schizonts on admission are shown in Table 1. Males were less likely to present with schizontaemia than female patients (Odds ratio 0.37 (95% confidence interval 0.20-0.67), p = 0.0018). Although the number of patients who acquired *P. falciparum* malaria outside of Africa was relatively low (n = 26), these patients tended to present with schizontaemia more frequently than patients who contracted malaria in Africa (Odds ratio 0.26 (95% CI 0.086-0.79), p = 0.0259). There were no differences between patients presenting with and without schizontaemia with regard to their immune status, ethnic background and use of malaria chemoprophylaxis, nor did they differ in duration of symptomatology. As detailed in Table 1, patients with schizontaemia presented more ill on admission: they had higher pulse rates, were more likely to have impaired consciousness and laboratory parameters were compatible with more severe degree of liver and kidney injury as well as more compromised tissue perfusion given the higher plasma lactate levels. Other laboratory parameters revealed that patients with schizontaemia also presented with significantly higher parasite loads, higher leucocyte counts, higher C-reactive protein levels and significantly lower platelet counts. When the WHO severity criteria for malaria [9] were assessed in both patient groups, patients with schizontaemia were more likely to present with or develop severe and complicated disease and they had to stay significantly longer in hospital than patients without demonstrable schizontaemia on admission (Table 2). Death occurred in two of 401 malaria patients, one with and one without schizontaemia on admission. Presence of schizontaemia on admission significantly increased with increasing parasite loads on admission (Chi square test for Trend, p < 0.0001), as shown in Figure 1.

**Table 1:** **General characteristics of patients with imported *Plasmodium falciparum *malaria grouped by presence or absence of schizontaemia on admission**

	**All patients**	**Schizontaemia**	**No schizontaemia**	**P value**
	**n = 401**	**n = 49**	**n = 352**	
**Demographic s**				
Age, years	39 (4–78)	41 (4–70)	39 (5–78)	NS
Male, female, n (%)	292 (72.8), 109 (27.2)	26 (53.1), 23 (46.9)	266 (75.6), 86 (24.4)	0.0018
**Continent of acquisition**				
Africa, n (%)	360 (89.8)	38 (77.6)	322 (91.5)	0.0196
Asia, n (%)	16 (4.0)	5 (10.2)	11 (3.1)	
South and Central America, n (%)	10 (2.5)	3 (6.1)	7 (2.0)	
Unknown, n (%)	15 (3.7)	3 (6.1)	12 (3.4)	
**Duration of signs/symptoms**				
<8 days, n (%)	259 (64.6)	30 (61.2)	229 (65.1)	NS
8-14 days, n (%)	74 (18.5)	15 (30.6)	59 (16.8)	
15-28 days, n (%)	27 (6.7)	0 (0)	27 (7.7)	
>28 days, n (%)	8 (2.0)	0 (0)	8 (2.3)	
Unknown, n (%)	33 (8.2)	4 (8.2)	29 (8.2)	
**Use of malaria chemoprophylaxis**				
No chemoprophylaxis, n (%)	272 (67.8)	35 (71.4)	237 (67.3)	NS
Inadequate use, n (%)	72 (18.0)	6 (12.2)	66 (18.8)	
Adequate use, n (%)	39 (9.7)	6 (12.2)	33 (9.4)	
Unknown, n (%)	18 (4.5)	2 (4.1)	16 (4.5)	
**Vital signs on admission**				
Body temperature, °C	38.5 (35.5-41.2)	38.7 (35.7-41.2)	38.5 (35.5-41.0)	NS
Pulse rate, beats per minute	95 (45–150)	105 (68–150)	92 (45–150)	0.0002
Systolic blood pressure, mm Hg	120 (64–190)	118 (64–160)	120 (73–190)	NS
Impaired consciousness (GCS < 15), n (%)	9 (2.2)	5 (10.2)	4 (1.1)	0.0019
Cerebral malaria (GCS ≤ 11), n (%)	5 (1.2)	2 (4.1)	3 (0.9)	NS
**Laboratory data on admission**				
Haemoglobin, mmol/L	8.3 (2.5-11.1)	7.8 (3.8-10.9)	8.4 (2.5-11.1)	NS
Leucocyte count, x 10^9^/L	5.1 (1.3-18.5)	6.1 (2.5-18.5)	5.0 (1.3-13.4)	0.011
Thrombocytes, x 10^9^/L	86 (2–385)	38 (10–164)	94 (2–385)	<0.0001
C-reactive protein, mg/L	98 (5–476)	166 (22–476)	87 (5–373)	<0.0001
Serum creatinine, μmol/L	95 (39–1,081)	110 (39–1,081)	95 (47–871)	0.0019
Serum sodium, mmol/L	135 (115–146)	132 (115–142)	135 (119–146)	<0.0001
Lactate dehydrogenase, U/L	275 (118–2,297)	435 (135–2,038)	262 (118–2,297)	<0.0001
Total bilirubin, μmol/L	24 (4–416)	44 (9–269)	23 (4–416)	<0.0001
Plasma lactate, mmol/L	1.6 (0.5-6.2)	2.7 (0.6-6.2)	1.5 (0.5-4.7)	<0.0001
**Parasite count**				
*P. falciparum* load (asexual parasites/μL)	8,400 (2–1,380,600)	162,000 (144–1,380,600)	5,430 (2–860,000)	<0.0001
Gametocytes, presence, absence, n (%)	64 (16.0), 337 (84.0)	11 (22.4), 38 (77.6)	53 (15.1), 299 (84.9)	NS

**Table 2:** **Outcome measures of patients with imported *Plasmodium falciparum *malaria, grouped by presence or absence of schizontaemia on admission**

	**All patients (n = 401)**	**Schizontaemia (n = 49)**	**No schizontaemia (n = 352)**	**P-value**	**Odds ratio (95% CI)**
**Case-fatalities, n (%)**	2 (0.5)	1 (2.0)	1 (0.3)	NS	
**Severe malaria, n (%)**	62 (15.5)	33 (67.3)	29 (8.2)	<0.0001	23.0 (11.3-46.6)
**ICU admission, n (%)**	92 (22.9)	38 (77.6)	54 (15.3)	<0.0001	19.1 (9.2-39.6)
**Exchange transfusion, n (%)**	40 (10.0)	26 (53.1)	14 (4.0)	<0.0001	27.3 (12.6-59.2)
**Renal replacement therapy, n (%)**	7 (1.7)	4 (8.2)	3 (0.9)	0.0053	10.3 (2.2-47.7)
**Mechanical ventilation, n (%)**	4 (1.0)	3 (6.5)	1 (0.3)	0.0063	22.9 (2.3-224.8)
**Time in hospital, days (range)**	5 (0–56)	7 (0–56)	5 (0–19)	<0.0001	

The diagnostic accuracy of schizontaemia as a predictor for severe malaria was characterized by a high specificity, negative predictive value and an acceptable positive predictive value. When compared to the performance of plasma lactate, schizontaemia had a lower sensitivity but a higher specificity and positive predictive value for severe disease. When compared to the performance of C-reactive protein, schizontaemia had a higher specificity and positive predictive value for severe disease (Table 3). Using binary logistic regression analysis, schizontaemia was found to be an independent predictor for severe malaria with odds ratios substantially exceeding that found for C-reactive protein and lactate but not that found for parasite load (Table 4).

**Table 3:** Descriptive statistics of schizontaemia, lactate and C-reactive protein as predictors for severe malaria

	**Schizontaemia (presence)**	**Plasma lactate (mmol/L)**	**C-reactive protein** **(mg/L)**
**Number**	401	240	381
**Median (range)**	N/A	1.6 (0.5-6.2)	98 (5–476)
**Mean**	N/A	1.9	111
**Standard deviation**	N/A	1.05	78
**Optimal cut-off**	N/A	≥1.7	≥142
**Sensitivity**	0.53 (0.40-0.66)	0.80 (0.67-0.89)	0.76 (0.63-0.86)
**Specificity**	0.95 (0.92-0.97)	0.63 (0.55-0.70)	0.75 (0.70-0.80)
**Youden’s index**	0.48	0.43	0.51
**PPV**	0.67 (0.52-0.80)	0.39 (0.30-0.49)	0.35 (0.27-0.45)
**NPV**	0.92 (0.88-0.94)	0.91 (0.85-0.95)	0.95 (0.91-0.97)

**Table 4:** Results of binary logistic regression analysis of explanatory variables for severe malaria

	**β**	**S.E. β**	**Wald’s χ**	**df**	**P value**	**Odds ratio (95% CI)**
**C-reactive protein**	0.01	0.04	8.326	1	0.004	1.01 (1.00-1.02)
**Log parasitaemia**	2.65	0.56	22.325	1	<0.001	14.13 (4.71-42.38)
**Plasma lactate**	0.42	0.27	3.564	1	NS	1.52 (0.89-2.57)
**Schizontaemia**	1.87	0.62	9.024	1	0.003	6.52 (1.92-22.13)
**Constant**	-16.63	2.93	32.133	1	<0.001	N/A

## Discussion

Sequestration of parasitized erythrocytes in the microvasculature of vital organs is a cardinal feature of *P. falciparum* malaria and is a cause of major pathology in severe disease [1-4]. Maturing *P. falciparum* parasites disappear from the peripheral blood after 24–26 hours to complete the asexual life cycle in the microvasculature, as the tendency to adhere to the endothelial wall increases during the maturation process [6]. This process of sequestration accounts for the fact that peripheral parasite counts do not always correlate well with the sequestered parasite burden. This discrepancy was noted soon after the use of the peripheral parasitaemia to predict the outcome of *P. falciparum* infection became common practice in the 1930s [5].

In the microvasculature, rupturing schizonts release up to 32 merozoites [7,16], causing an exponential rise in parasitaemia. A high schizont count is therefore likely to precede a rise in parasitaemia and might be an early marker for severe disease. The presence of schizontaemia may also be relevant to guide selection of parenteral treatment. As compared to quinine, artemisinin derivatives like artesunate have an anti-malarial efficacy to the broadest spectrum of parasite stages, including the generally less drug-sensitive schizonts [1,7]. This may provide another argument to treat patients with schizontaemia with artemisinin derivatives given the convincing proof of past studies in regions of malaria endemicity showing that artesunate was superior over quinine for treatment of severe malaria in both children and adult patients [17,18], not only in terms of reduction of mortality and parasite clearance but also in ease of use. Recent studies in non-endemic industrialized regions confirmed that in non-immune travellers with imported malaria, treatment with artesunate results in a comparable rapid parasite clearance to that observed in semi-immune individuals [19,20].

In the present study schizontaemia was found to be a useful marker for severe malaria. Although schizontaemia was more frequently seen in patients with high-grade parasitaemia, the predictive power appeared to be independent of parasitaemia. In the study by Silamut and White [6] in adult Thai patients and children from The Gambia, it was demonstrated that a predominance of mature parasite forms in the peripheral blood reflect a greater sequestered parasite mass and the presence of >10 ^4^ mature trophozoites and schizonts per μL of peripheral blood was found to have a high sensitivity (90%) and specificity (70%) for fatal outcome. The data of the present study confirm the prognostic value of schizontaemia for severe disease in predominantly non-immune travellers, comparable to that observed in semi-immune patients in region of malaria endemicity. However, it should be noted that besides differences in presumed immunity towards malaria, the current study also differed in its design with regard to outcome. In many studies in malaria-endemic regions, the primary outcome measure is usually survival or death and studies are restricted to these severe cases using the WHO severity criteria as an entry criterion. Since case fatalities are less common in the western non-endemic, resource-rich setting [12] and malaria is only seen as a sporadic imported disease, the present study in patients with imported malaria focused on severe malaria as the primary outcome, using the WHO criteria to define disease severity.

Unfortunately, for the present study quantitative counts of the different parasite stages were not available. Although quantification would likely have resulted in a more precise estimation of its prognostic value, the specificity of schizontaemia was found to be a useful tool for exclusion of severe disease. The negative predictive value of schizontaemia is high, and comparable to that of C-reactive protein and lactate. Moreover, unlike C-reactive protein and lactate [13-15] schizontaemia on admission was also associated with a high positive predictive value. This may provide the clinician with a helpful tool for decision making in the acute care setting by allowing an early identification of those malaria patients who are at risk of a more severe and complicated course than would normally be expected when considering peripheral parasitaemia alone.

## Conclusion

Absence of schizonts on admission in travellers with imported *P. falciparum* malaria was found to be a specific marker for exclusion of severe disease; in contrast, presence of schizonts had a high positive predictive value for presence of severe disease and can thus help to identify patients that may be candidates in need of intensive monitoring and timely administration of preferably (parenteral) artemisinin derivatives.

## Competing interests

The authors declare that they have no competing interests.

## Authors’ contributions

MEvW contributed to the data acquisition and analysis and writing of the manuscript. MdeMM, KVJ and JvH participated in the data analysis and revising of the manuscript. RK is responsible for collection of patient materials, patient data and database management. PJvG designed the study, participated in the data acquisition and analysis and in writing and revising the manuscript. All authors have read and approved the final version.
